# Midgut and Head Transcriptomic Analysis of Silkworms Reveals the Physiological Effects of Artificial Diets

**DOI:** 10.3390/insects13030291

**Published:** 2022-03-15

**Authors:** Juan Li, Chunbing Chen, Xingfu Zha

**Affiliations:** 1 State Key Laboratory of Silkworm Genome Biology, Southwest University, Chongqing 400715, China; worker@swu.edu.cn (J.L.); chenchunbingxxc@163.com (C.C.); 2 School of Life Sciences, Southwest University, Chongqing 400715, China

**Keywords:** artificial diet, silkworm, midgut, head, transcriptome

## Abstract

**Simple Summary:**

As oligophagous insects, silkworms have a very limited diet. Here, the transcriptomic characteristics of the head and midgut tissues of fifth instar male and female silkworms were studied to elucidate the effects of different food structures on silkworm innate immunity. Compared with mulberry leaves, artificial diets decreased the expression levels of immune-associated genes, thus decreasing silkworm immunity and susceptibility to pathogen infections.

**Abstract:**

Silkworms, a model lepidopteran insect, have a very simple diet. Artificial diets as an alternative nutrient source for silkworms are gradually being developed. To understand the effects of various nutrients on the growth and development of silkworms, we studied the transcriptomic differences in the midgut and head tissues of male and female silkworms fed either fresh mulberry leaves or artificial diets. In the artificial diet group, compared with the control group (fed mulberry leaves), 923 and 619 differentially expressed genes (DEGs) were identified from the midgut, and 2969 and 3427 DEGs were identified from the head, in female and male silkworms. According to our analysis, the DEGs were mainly involved in the digestion and absorption of nutrients and silkworm innate immunity. These experimental results provide insights into the effects of different foods, such as artificial diets or fresh mulberry leaves, on silkworms.

## 1. Introduction

The silkworm (*Bombyx mori*), a model insect for the study of lepidopteran species, has been domesticated for silk production and has a long history of breeding [1]. In addition, the silkworm pupa can be used in food and medicine applications because of its rich nutritional content [2]. Silkworms are oligophagous insects whose only food is mulberry leaves (ML), from which they derive nutrients and water [3]. ML grow best in the spring and contain higher nutrients at that time; because the quality of ML is poor in the autumn and winter, the development of silkworms in spring is better than that in autumn and winter [4]. As ML are a seasonal food susceptible to pests, an artificial diet (AD) for silkworms has been developed, which can be mass-produced and supplemented with nutrients as needed [5,6]. In addition to solving the seasonal problem of mulberry leaves, AD can also improve silk production by modifying the composition of artificial diet [5]. From the 1980s to the 1990s in Japan, silkworms were cultivated on ADs on a large scale. However, silkworm rearing on artificial diet remains only in the laboratory stage, and is far from achieving universal application.

The nutrients obtained by silkworms mainly come from mulberry leaves, and the digestion of silkworms on mulberry leaves is inseparable from the midgut. The midgut of insects plays an important role in food digestion and nutrient absorption. In addition, the midgut, an immune organ, is the first line of defense against pathogenic microorganisms [7], and it acts as a defense against some pathogens that are infected through the oral cavity [8]. This series of actions is maintained by the non-cellular structure of the insect peritrophic membrane secreted by the midgut epithelial cells [9]. There is also a complex interaction between gut tissue and the brain. For example, gut microbes in mice are involved in the regulation of neurotransmitter production in the brain and also affect brain myelination patterns [10,11]. In insects, the head is the most complex structure, which can control development, movement, feeding and other behaviors, and in the silkworm, the head also has the function of spinning.

Understanding the nutritional content of ML is essential to produce an AD meeting silkworms’ needs. The components of ML that can be used by silkworms mainly include protein, carbohydrates, lipids, inorganic substances, moisture and vitamins [12,13,14]. In the artificial diet, researchers can improve the content of nutrients as needed. Although the main component of the AD remains ML, the metabolic capacity of silkworms remains poorer on ADs than ML: silkworms fed ADs have low silk yield, slow larval growth and development, and low efficiency of silk protein synthesis [15]. Differences in the proteome and fecal metabolome of silkworm pupae fed ML vs. an AD have been observed, thus affecting lipid transport and metabolism in silkworm pupae treated with various nutrients. The content of amino acids, carbohydrates and fatty acids in metabolites in silkworms fed an AD is significantly diminished [16,17]. The silkworm midgut not only serves as the site of nutrient digestion and absorption, but also helps resist pathogenic microorganisms. However, little is known regarding the transcriptomic differences between the midgut and head of silkworms reared on ADs vs. ML. Some questions that must be answered are: (1) What are the sex-specific transcriptomic differences in silkworms reared on AD vs. ML? (2) What are the transcriptomic differences between the midgut and head in silkworms reared on an AD vs. ML? (3) At the transcriptional level, how can the effects of different nutrients on silkworms be explained? To answer these questions, we analyzed transcriptomic differences in the midgut and head tissues of male and female silkworms fed ML and an AD.

## 2. Materials and Methods

### 2.1. Preparation of ML and AD

The fresh ML were provided by the Mulberry Garden, Southwest University. The AD consists of 20% mulberry leaf powder, 30% soybean meal, 40% corn meal, 2% citric acid, 0.9% vitamin B complex, 3.3% carrageenan, 0.3% sorbic acid, 0.5% of gallic acid, 1% ascorbic acid and 2% mixed inorganic salts. The AD mixture was added to ddH_2_O according to the mass ratio (1:1.5), and after mixing, heated at 121 °C for 20 min, and stored at 4 °C after the treatment.

### 2.2. Rearing of Silkworms

The silkworms (*Dazao*) were provided by the State Key Laboratory of Silkworm Genome Biology (Southwest University, Chongqing, China). The silkworm larvae were fed fresh ML or AD under standard conditions, at a temperature of 25 ± 2 °C under a photoperiod of 12 h light/12 h dark with 75% humidity. When the silkworms reached the fifth instar, the larvae were sexed according to their morphological features. The presence of four opalescent genital stalens at the 8, 9 abdominal ganglia line were considered to identify females, whereas the presence of only one genital stalen was used to identify males [18]. The reason for the difference in the growth rates between female and male silkworm larvae may be that the utilization of nutrients in the midgut varies with sex [19]. We fixed the silkworm larvae (on the third day of the fifth instar) on the dissection plate, then took out the midgut, discarded the food waste in the midgut, cut the middle part of the midgut, and cut the head tissue at the same time. The samples were frozen in liquid nitrogen and stored at −80 °C. Three replicates were collected for each set of samples.

### 2.3. RNA Extraction, cDNA Library Preparation and Sequencing

Total RNA from eight samples (three biological replicates) were extracted with a TRIzol reagent kit according to the manufacturer’s protocol. First, RNA degradation and contamination were monitored on 1% agarose gels. Then, a NanoPhotometer^®^ spectrophotometer (IMPLEN, Westlake Village, CA, USA) was used to verify the RNA purity, and an RNA Nano 6000 Assay Kit for the Bioanalyzer 2100 system (Agilent Technologies, Santa Clara, CA, USA) was used to assess RNA integrity. A total amount of 1 µg RNA per sample was used as input material for the RNA sample preparations. Sequencing libraries were generated with a NEBNext^®^ UltraTM RNA Library Prep Kit for Illumina^®^ (NEB, Ipswich, MA, USA) according to the manufacturer’s recommendations, and index codes were added to attribute sequences to each sample. Samples were barcoded, multiplexed and sequenced (150 bp paired-end) with the Illumina Novaseq platform.

### 2.4. Transcriptome Data Quality Controls and Alignment

To obtain reliable data, we first processed raw data (raw reads) in fastq format through in-house perl scripts. In this step, clean data (clean reads) were obtained through the removal of reads containing adapters, reads containing poly-N and low quality reads from raw data. Simultaneously, the Q20, Q30 and GC content in the clean data were calculated. All downstream analyses were based on the clean data with high quality. The silkworm genome SilkBase (http://silkbase.ab.a.u-tokyo.ac.jp/cgi-bin/index.cgi) (accessed on 16 July 2019) was used as a reference. Reference genome and gene model annotation files were downloaded from the genome website directly. The index of the reference genome was built with Hisat2 v2.0.5, and paired-end clean reads were aligned to the reference genome with Hisat2 v2.0.5 (4). All raw transcriptome data have been submitted to the National Centre for Biotechnology Information SRA database (PRJNA729897).

### 2.5. Bioinformatic and Computational Analysis

The Pearson’s correlation calculation method was used to evaluate the correlation between samples. The expected number of fragments per kilobase of transcript sequence per million base pairs sequenced (FPKM) was calculated with StringTie software and is the most commonly used method for estimating gene expression levels. Differentially expressed gene (DEG) lists based on comparative analysis were identified with DESeq2 software, and genes with a *p*-value <0.05 were considered differentially expressed. The *p* values were adjusted with the Benjamini–Hochberg method. A corrected *p*-value of 0.05 and absolute fold change of 2 were set as the threshold for significant differential expression [20,21,22]. Selected genes were subjected to Gene Ontology (GO) (https://wego.genomics.cn/) (accessed on 12 September 2019) enrichment analysis with the clusterProfiler R package, and GO terms with a corrected *p*-value <0.05 were considered significantly enriched in DEGs. The Kyoto Encyclopedia of Genes and Genomes (http://www.genome.jp/kegg/) (accessed on 12 September 2019) was used to understand the genes’ functions.

### 2.6. RNA Reverse Transcription and qRT-PCR of DEGs

By using the SilkBase database, we first found the CDS sequences of immune-related differentially expressed genes, and then designed the corresponding primers for quantitative real-time PCR (qRT-PCR) according to the CDS sequences, and the primer sequences were provided in the supplementary material. According to the manufacturer’s instructions (Total RNA Kit, OMEGA, Shanghai, China), we extracted total RNA from head and midgut tissue of day-3-fifth-instar silkworms, reverse transcribed the total RNA with a reverse transcription kit (Cat. No. RR047A, Takara, Dalian, China), and diluted the cDNA template concentration to 200 ng/μL, and performed qPCR reaction with the SYBR^®^ Premix Ex Tag™ kit. Each reaction system is as follows: 20 μL SYBR, 0.8 μL F-terminal primer, 0.8 μL R-terminal primer, 0.4 μL Dye II, 6 μL ddH_2_O, 2 μL template. Data were processed using 2^−^^ΔΔCt^ calculation formula [23].

## 3. Results

### 3.1. Phenotypic Observations and Sequencing Data

Both the experimental group and the control group were reared at indicated temperature and humidity. In the fifth instar, we measured the body length of 20 silkworms randomly selected from the AD group and the ML group, respectively. The average body length of the AD group was 48.5 mm, whereas 46.4 mm in the ML group (Supplementary Figure S1). The results indicated that the body size in the experimental group was slightly larger than that in the control group (Supplementary Figure S2). The probable reason why the silkworms fed with AD grew slower, but were larger than those in the control group was that the silkworms fed with AD had a longer instar duration and received more food. Reads were obtained from two tissues (midgut and head) in the ML and AD treatment groups. The highest number of raw reads (52,525,801; Table 1) was found in the male silkworm head (MH) tissue in the AD group. The raw reads from the female silkworm midgut (FG) tissue were higher (48,376,351) than other samples in the ML group. After filtering the original data and quality testing, the number of clean reads was 47,594,289 and 51,984,274, respectively. These clean reads of 24 samples were then mapped to reference genome sequences from the Silkworm Genome Database. In the ML group, the highest average clean reads relative to raw reads accounted for 98.79% in the female silkworm head (FH) tissue. In the AD group, the highest proportion of clean reads relative to raw reads was observed in the MH tissue, reaching 98.97%. The longest total average length of the screened sequence was 7.14 G and 7.8 G, respectively. The lowest Q20 and Q30 of these samples was 95.74% and 89.22%, thus indicating that the sequencing data were suitable for further analysis.

### 3.2. Global Differential Gene Expression Changes

**Figure 1:**
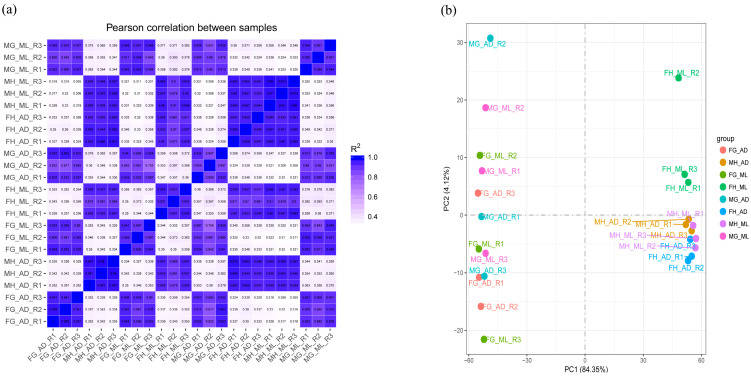
Correlation analysis and PCA. Correlation analysis displayed in (**a**) distance heatmap, with a legend representing Pearson correlation coefficient values between samples, and (**b**) principle component analysis based on data counts from RNA-Seqs prepared in triplicates for FG_AD, MH_AD, FG_ML, FH_ML, MG_AD, FH_AD, MH_ML, MG_ML.

Principal component analysis (PCA) and distance heat maps were used to evaluate the correlation between tissues in the two groups to understand the changes in transcriptome levels. The two groups of treatment included feeding on ML and an AD. The tissues included FH, FG, MH and male silkworm midgut (MG). We performed experiments on three independent replicates per group. The correlation between different replicates receiving the same treatment and the same organization showed a tight clustering pattern (Figure 1). According to the PCA plot, these patterns were mainly driven by the tissue (PC1 = 84.35%), followed by the two dietary treatments (PC2 = 4.12%).

**Figure 2:**
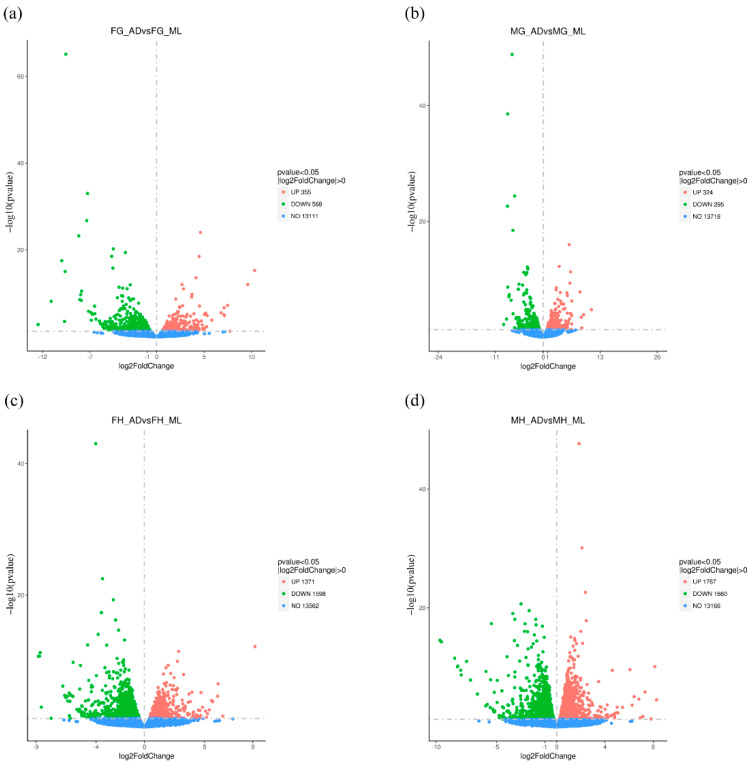
Volcano plot of DEGs in silkworm fed AD and ML. Volcano plot for pair wise comparison for (**a**) FG_ADvsFG_ML, (**b**) MG_ADvsMG_ML, (**c**) FH_ADvsFH_ML and (**d**) MH_ADvsMH_ML. Horizontal oridinate represents the fold change of gene expression, and the vertical ordinate represents the statistical significance of the change. Number of DEGs expressions (normalized to Log2 fold change) are displayed on the right side of each picture based on a *p*-adj cut-off of <0.05. The red plot represents a differentially upregulated gene, a green plot represents a differentially downregulated gene, and the blue plot means a gene that does not show differential expression.

DEGs were genes whose expression levels were statistically significantly different under different treatment conditions. On the basis of correlation analysis, DEGs were identified from different tissues under different diet treatments. We observed no significant difference in the number of DEGs between FG_AD/FG_ML and MG_AD/MG_ML, and between FH_AD/FH_ML and MH_AD/MH_ML. With volcano plots, a total of 923 and 2969 DEGs were identified for FG_AD/FG_ML and FH_AD/FH_ML (Figure 2); meanwhile, a total of 619 and 3427 DEGs were identified for MG_AD/MG_ML and MH_AD/MH_ML. The comparative forms of DEGs between midgut and head were presented in Supplementary Figure S3. The difference in the number of DEGs might reflect a series of synergistic processes. For example, under different nutritional conditions, the silkworm body metabolism and its innate immune ability will differ, thus, affecting the development of silkworm tissues and organs, and potentially silk production and reproduction ability. The effects of different diets on the silkworm heads were significantly greater than those on the midgut, among which the effects on the MH were greater than those on the FH, and the effects on the FG were greater than those on the MG.

**Figure 3:**
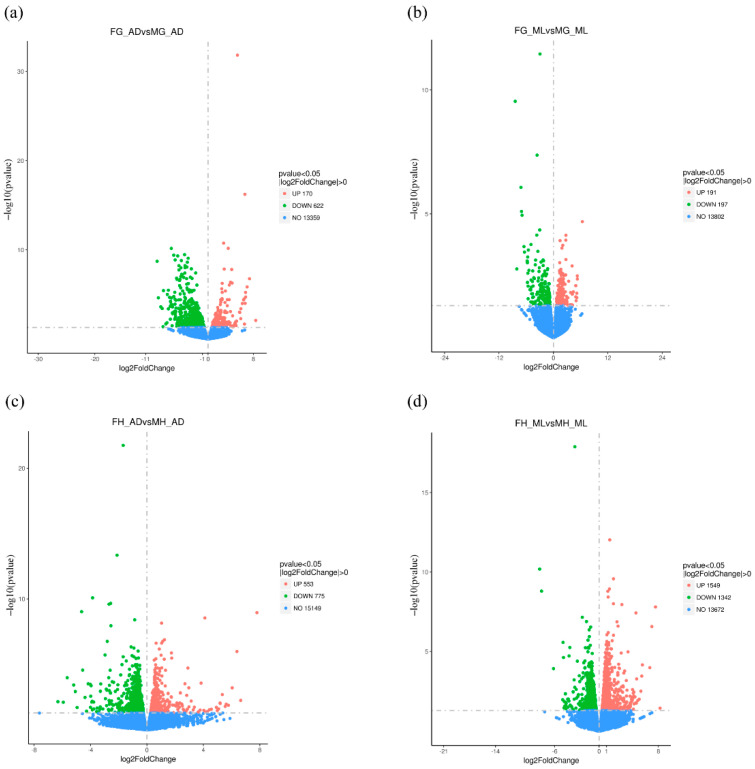
Volcano plot of DEGs between female and male silkworms. Volcano plot for pair wise comparison for (**a**) FG_ADvsMG_AD, (**b**) FG_MLvsMG_ML, (**c**) FH_ADvsMH_AD and (**d**) FH_MLvsMH_ML. Horizontal oridinate represents the fold change of gene expression, and the vertical ordinate represents the statistical significance of the change. Number of DEGs expressions (normalized to Log2 fold change) are displayed on the right side of each picture based on a *p*-adj cut-off of <0.05. The red plot represents a differentially upregulated gene, a green plot represents a differentially downregulated gene, and the blue plot means a gene that does not show differential expression.

In order to address the differences of transcription levels of AD- and ML-rearing silkworms between the sexes, we analyzed the DEGs of male and female silkworms under different dietary conditions. Based on the volcano plot, we found that in the midgut tissue, under AD feeding, there were 792 DEGs between the sexes (170 up-regulated and 622 down-regulated) (Figure 3), while in the case of ML feeding, there were only 388 DEGs (191 up-regulated and 197 down-regulated). In the head tissue, under AD feeding, there were 1328 DEGs between the sexes (553 up-regulated and 775 down-regulated), and under ML feeding conditions, there were 2891 DEGs (1549 up-regulated and 1342 down-regulated). The head is the control center of individual organisms, whose differences probably lead to the differences of individual development and behavior between the sexes. Our results showed that there were more DEGs between the sexes in silkworm head than that in the midgut.

### 3.3. Genes Associated with Digestion and Absorption of Nutrients

**Figure 4:**
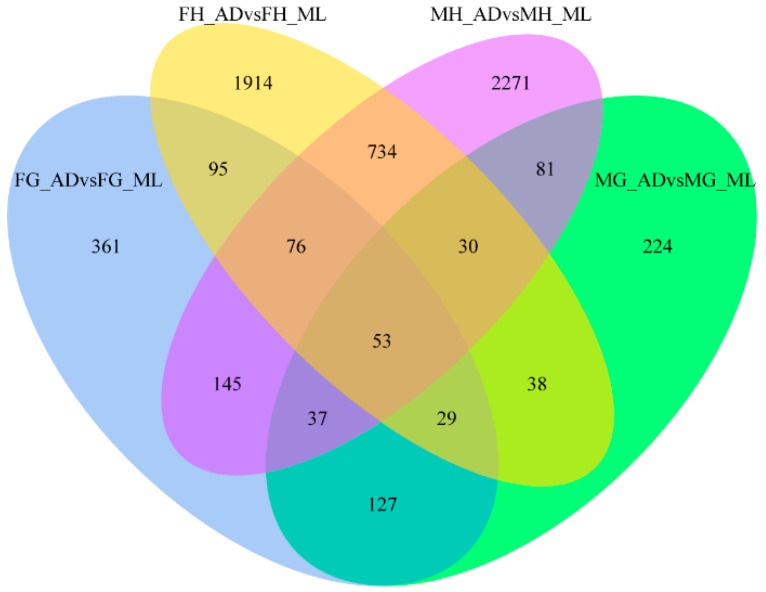
Venn diagram of DEGs. Venn diagram illustrating unique and common DEGs in FG_ADvsFG_ML, MG_ADvsMG_ML, FH_ADvsFH_ML and MH_ADvsMH_ML based on statistical significance cut-off *p*-adj <0.05.

**Figure 5:**
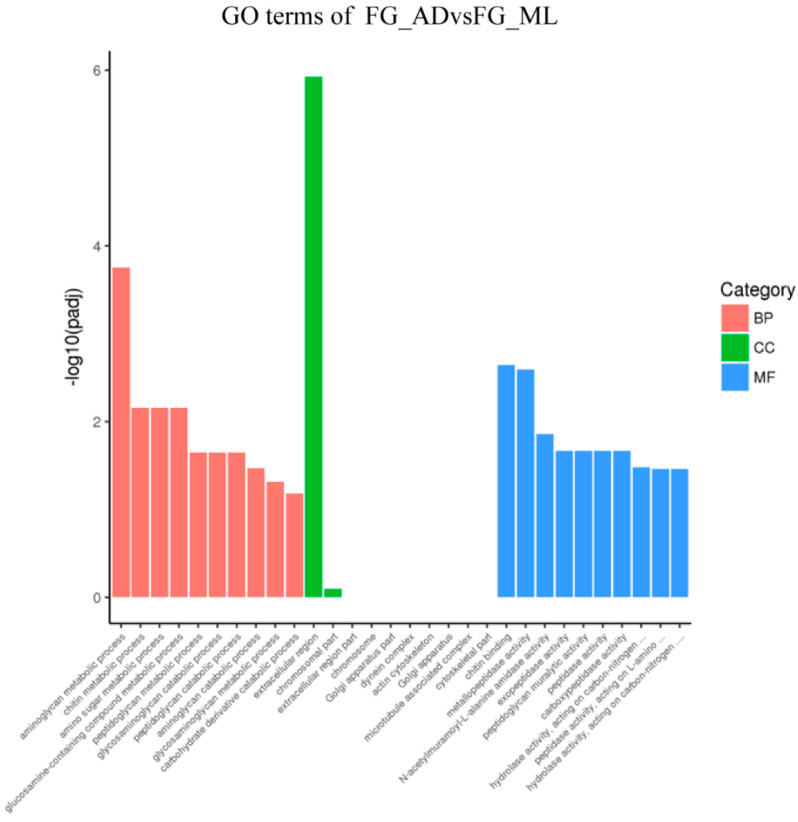
GO enrichment of FG_ADvsFG_ML. The differentially expressed genes were mainly related to biological processes (BP) such as aminoglycan metabolic process, chitin metabolic process, amino sugar metabolic process and glucosamine-containing compound metabolic process; cell components (CC) only related to extracellular region and chromosomal part, and the molecular functions (MF) such as chitin binding, metallopeptidase activity, N-acetylmuramoyl-L-alanine amidase activity.

**Figure 6:**
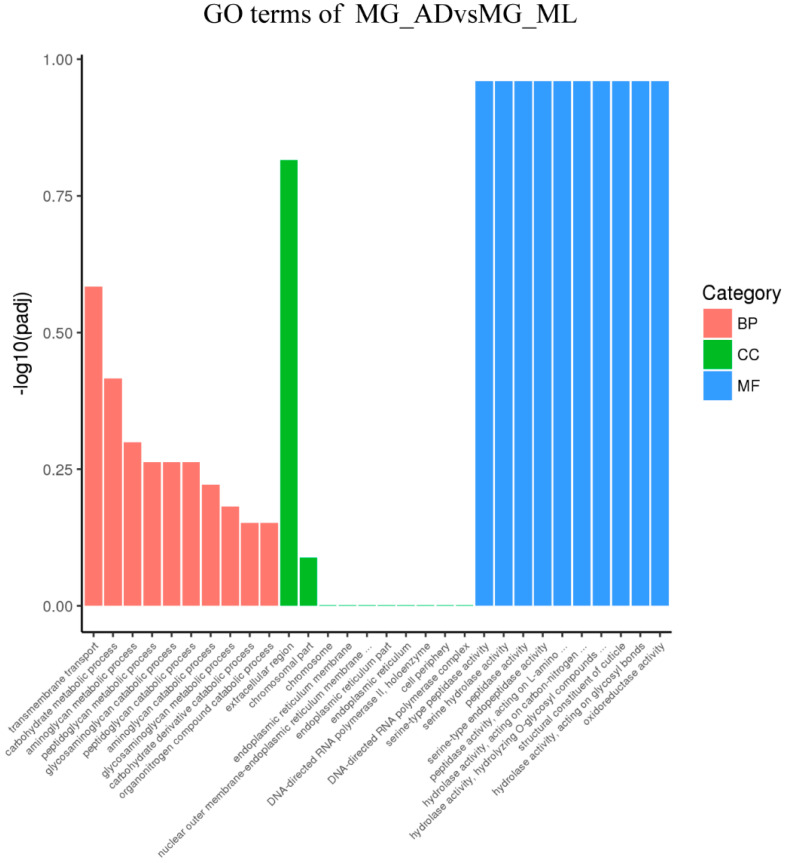
GO enrichment of MG_ADvsMG_ML. The differentially expressed genes were mainly related to biological processes (BP) such as transmembrane transport, carbohydrate metabolic process, aminoglycan metabolic process; cell components (CC) only related to extracellular region and chromosomal part, and the molecular functions (MF) such as serine-type peptidase activity, serine hydrolase activity and peptidase activity.

To better analyze the functions of DEGs and the related pathways in organisms, we performed GO enrichment and KEGG analysis on the DEGs detected in this comparision. First, DEGs from different tissues were organized into a Venn diagram to eliminate gene redundancy. The chart indicated 53 DEGs common across the four comparisons (Figure 4). Simultaneously, 361, 1914, 2271 and 224 uniquely expressed genes were found for the four comparisons. Most DEGs were specifically expressed in different tissues, whereas the commonly expressed DEGs might be important for silkworms’ digestion and absorption of nutrients. The GO enrichments from the FG_AD/FG_ML and MG_AD/MG_ML comparisons showed that the DEGs were mainly involved in biological processes such as aminoglycan metabolic process, transmembrane transport, carbohydrate metabolic process, peptidoglycan metabolic process and organonitrogen compound metabolic process (Figure 5 and Figure 6). Interestingly, among the enriched GO terms, metabolic and catabolic process were the most significant. The enriched cell components mainly involved the extracellular region and chromosomal region. The molecular functions were mainly chitin binding and serine-type peptidase activity. The enrichments for the FH_AD/FH_ML and MH_AD/MH_ML comparisons showed that the DEG functions were mainly translation, ribosome, and structural constituent of ribosome. Organonitrogen compound, peptide and amide biosynthetic process functions were identified in the FH_AD/FH_ML or MH_AD/MH_ML comparisons. All these biological processes are associated with the digestion and absorption of nutrients obtained by silkworms.

**Figure 7:**
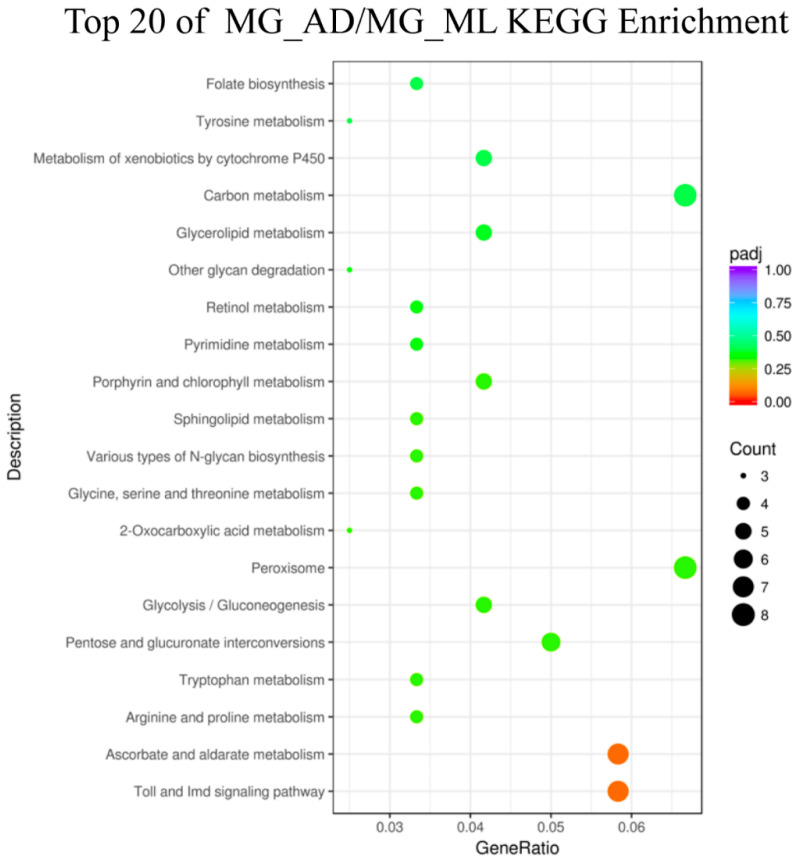
KEGG enrichment of MG_ADvsMG_ML. Statistics of top 20 pathway enrichment of differentially expressed genes in each pairwise. Padj is ranging from 0 to 1, and less value means greater intensiveness. The size of the plot represents the number of DEGs involved in the relevant pathway.

**Figure 8:**
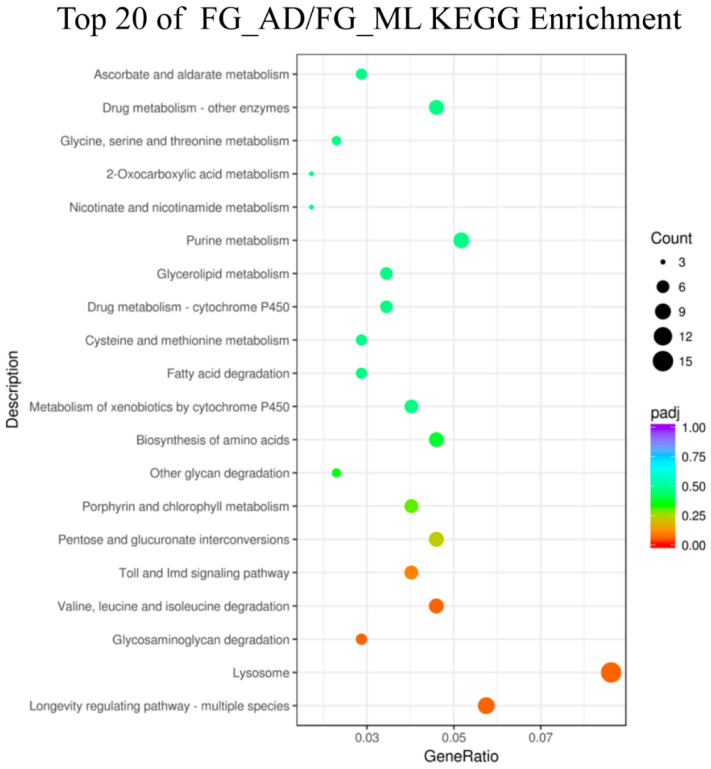
KEGG enrichment of FG_ADvsFG_ML. Statistics of top 20 pathway enrichment of differentially expressed genes in each pairwise. Padj is ranging from 0 to 1, and less value means greater intensiveness. The size of the plot represents the number of DEGs involved in the relevant pathway.

The KEGG database was used to analyze the pathways of the DEGs in the comparisons of FG_AD/FG_ML, MG_AD/MG_ML, FH_AD/FH_ML, and MH_AD/MH_ML. The largest numbers of genes were annotated with lysosome, carbon metabolism and ribosome functions. We identified 92 pathways in the comparison of FG_AD/FG_ML, for which the lysosome pathway had the largest number of genes: ten upregulated and five downregulated. In the experimental group of MG_AD/MG_ML (Figure 7), a total of 84 pathways were involved, and the largest numbers of DEGs were associated with the Toll and Imd signaling pathway (one upregulated gene among seven total genes) and ascorbate and aldarate metabolism (five upregulated genes among seven total genes). A total of 112 pathways were identified for FH_AD/FH_ML (Figure 8). Interestingly, among pathways, the genes with ribosome (92 downregulated genes among 92 total genes) and oxidative phosphorylation (58 downregulated genes among 58 total genes) functions were all downregulated. In contrast, for MH_AD/MH_ML, the genes with ribosome (66 upregulated genes among 67 total genes) and protein processing in the endoplasmic reticulum (56 upregulated genes among 62 total genes) pathway were almost all upregulated.

### 3.4. Genes Associated with Innate Immunity

**Figure 9:**
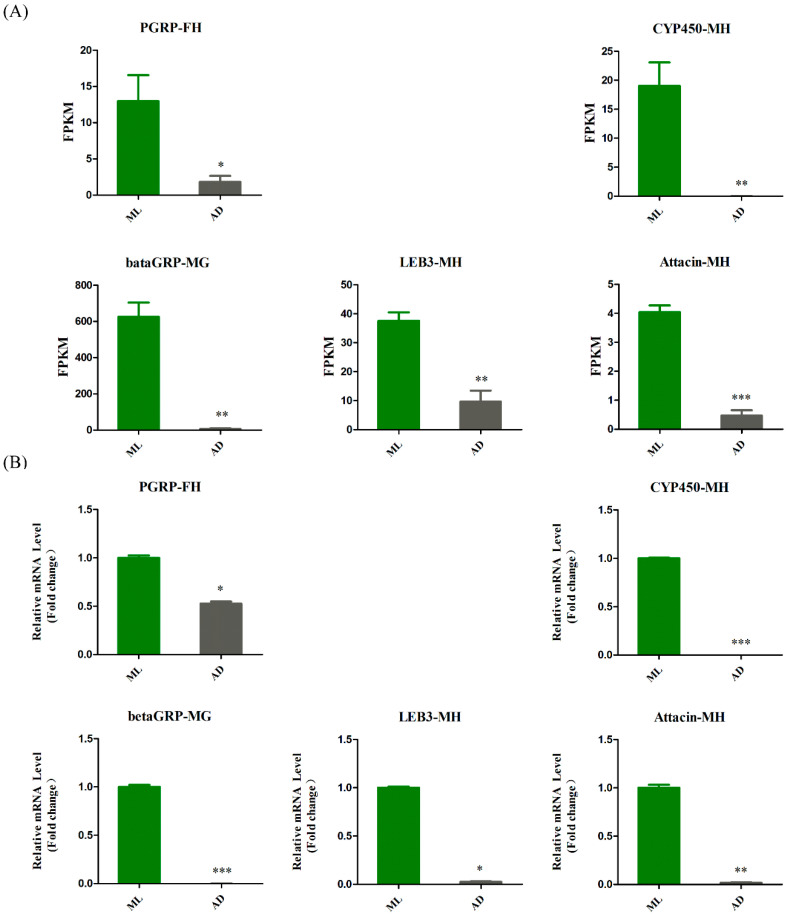
Analysis of differentially expressed genes associated with innate immunity. The expression level of differentially expressed genes was measured by FPKM value (**A**) and qRT-PCR (**B**), ML represented the control group and AD represented the experimental group. The above five congenital immune-related genes were significantly reduced in the experimental group. The student’s *t*-test was used to evaluate the statistical differences between the samples. A different number of asterisks indicates a difference in significance. (* *p*-value < 0.05, ** *p*-value < 0.01, *** *p*-value < 0.001).

To better understand the differences between the experimental group and the control group, we analyzed the genes with significant expression differences in the four groups. Interestingly, most of the genes with significant differences were associated with the innate immune pathway. We then selected several representative genes and constructed a histogram according to FPKM values (Figure 9A). The expression of all these genes was significantly diminished in the experimental group. At the same time, we also verified the expression levels of differentially expressed genes in the experimental group and the control group by qRT-PCR, and the results showed the same trend as the FPKM map (Figure 9B), thus indicating that the transcriptome data are reliable. 

## 4. Discussion

Different sources of nutrients will have different effects on individual organisms. Although AD can be formulated according to the needs of the purpose, they will also have a certain impact on the absorption and metabolism of nutrients in silkworms. For example, the expression of related genes in the glycolysis/gluconeogenesis pathway was affected in silkworms fed AD, and the expression of a hexokinase gene (*Hex-t2*), which catalyzes the first step of glucose metabolism, was decreased (Supplementary Figure S4). Similarly, all differentially expressed genes related to various type of N-glycan biosynthesis were downregulated and thus the activity of this pathway was probably decreased. In contrast, the expression of DEGs involved in ascorbate and aldarate metabolism, arginine and proline metabolism, tryptophan metabolism and pentose and glucuronate interconversions pathways was up-regulated, indicating that AD-rearing silkworms can promote some metabolic processes. 

Silkworms reared on AD vs. ML showed sex-specific transcriptomic differences. Among the top-five enriched pathways for DEGs, neuroactive ligand-receptor interaction, and pentose and glucuronate interconversions had sex differences in the midgut of silkworms reared on AD vs. ML (Supplementary Figure S5). Meanwhile, two enriched pathways of ribosome and oxidative phosphorylation had sex differences in the head of silkworms reared on AD as well as ML (Supplementary Figure S5). These results provided some clues to study on sex differences in the silkworm.

The Toll and Imd signaling pathway are responsible for the immune response through the production of antimicrobial peptides. A key member of this pathway is PGRP, a peptidoglycan-hydrolyzing amidase that recognizes the peptidoglycans in Gram-positive or Gram-negative bacteria. PGRP (peptidoglycan recognition protein) plays an important role in the balance between the immune system’s protecting beneficial microorganisms and eliminating pathogens. Studies have shown that different PGRP genes perform different functions. For example, PGRP-SA recognizes peptidoglycans from fungi and Gram-positive bacteria and activates Toll receptors, whereas PGRP-LC and PGRP-LE mainly induce the production of antimicrobial peptides targeting Gram-negative bacteria [24,25]. PGRP-LB, another member of the PGRP family, degrades peptidoglycan produced by Gram-negative bacteria and down-regulates the Imd pathway, thus, controlling the immune response of flies to bacteria in the gut [26,27]. Previous studies have isolated PGRP protein from the hemolymph of *Bombyx mori* [28]. *Enterococcus mundtii* (*E. mundtii*) is a pathogenic bacterium that causes silkworm flacherie disease, which usually occurs in silkworms fed an AD. This pathogen has been isolated from fresh ML or AD made from ML. When silkworms are infected with this pathogen, the larvae can become anorexic, thus affecting their development [29,30]. Among DEGs, the expression level of PGRP in the AD group was hundreds of times (KWMTBOMO09651: 295.69-fold down-regulation in the FG group and 291.39-fold down-regulation in the MG group) lower than that in the ML group, thus, indicating that, compared with that in the control group, the expression of the PGRP gene in the midgut in silkworms fed AD was significantly diminished; therefore, the antimicrobial ability of silkworms in the experimental group was significantly weakened.

Cytochrome P450 (CYP450), a superfamily of heme-thiolate proteins, is involved primarily in the metabolism of endogenous and exogenous substances, such as pheromones and insecticides. Previous studies have shown that the CYP families contain 70 P450 gene superfamilies [31,32]. Experiments on insecticide treatments in bees have shown that the immune response activates the CYP9E2 gene, which has a detoxification function that is active against bacterial infection [33]. In FH and MH, the expression level of P450 (KWMTBOMO10600) in the AD group was almost zero, but that in the ML group, the level exceeded 100. The different expression levels of these CYP genes indicated that individuals in the experimental group were less able to mount an immune response to pathogenic bacteria or pesticides. Moreover, the metabolic capacity of silkworms toward internal and external substances in the experimental group rapidly weakened.

The β-1,3-glucan recognition protein (betaGRP) specifically recognizes β-1,3-glucan, a fungal cell wall component, mainly by recognizing its triple helix structure. This recognition activates a prophenoloxidase cascade and the Toll signaling pathway, thus triggering innate immunity to eliminate pathogens [34,35]. Recent studies have shown that the transcript levels of the βGRP gene are significantly upregulated when insects are infected with Escherichia coli and Staphylococcus aureus [36]. BetaGRP then recognizes and binds bacteria, thus defending against invading pathogens. Significant differences were detected in βGRP-1 and βGRP-2 gene expression in the FG and MG; the expression level of the βGRP (KWMTBOMO06450) gene in the AD group was 100 times lower than that in the ML group. These results indicated that the innate immunity in the experimental group was far inferior to that of the control group.

Antimicrobial peptides are important components of innate immunity in insects. Several major types of antimicrobial peptides include attacin, cecropin, moricin, gloverin and lebocin [37]. Lebocin (KWMTBOMO05616), an antimicrobial peptide in lepidopteran insects, is significantly up-regulated in the midgut during metamorphosis and is part of a multi-gene family [38]. The expression of this gene is induced by bacterial injection in silkworms [39,40,41]. Previous studies have shown that BmBR-C Z4 and BmEts enhance the promoter activity of this gene and up-regulate the expression of antimicrobial peptides, thus protecting silkworms against infection by pathogenic microorganisms [42,43]. On the basis of our DEG results, we hypothesized that AD feeding may decrease the expression of antimicrobial peptide genes, thus affecting the immune response of silkworms.

Attacin (KWMTBOMO03709), another antimicrobial peptide gene, has clear antimicrobial activity against *Escherichia coli*. In addition, after infection by *E. coli*, *Beauveria bassiana*, *Micrococcus luteinus*, and nuclear polyhedal virus, the expression of this gene in the fat body in silkworms significantly increases [44,45], and the induction effect of this gene is strongest toward bacteria after UV inactivation [46]. Compared with that in the control group, the expression level of this gene in the experimental group was significantly down-regulated among the DEGs; therefore, our study indicated that AD feeding significantly decreases silkworm innate immunity.

## Figures and Tables

**Table 1:** Basic information of reads.

Category	ML				AD			
Parameter	FG	FH	MG	MH	FG	FH	MG	MH
raw reads	48,376,351	45,283,683	44,425,225	46,127,387	46,232,769	40,543,023	42,918,998	52,525,801
clean reads	47,594,289	44,735,653	43,798,904	45,544,796	45,407,637	40,039,759	42,469,478	51,984,274
clean bases (bp)	7.14 (G)	6.71 (G)	6.57 (G)	6.83 (G)	6.81 (G)	6.01 (G)	6.37 (G)	7.8 (G)
clean reads/raw reads	0.9838	0.9879	0.9859	0.9874	0.9822	0.9876	0.9895	0.9897
Q20 (%)	96.09	95.74	95.98	95.97	95.92	95.91	95.84	96.26
Q30 (%)	89.98	89.22	89.71	89.68	89.64	89.56	89.53	90.33
GC (%)	47.83	44.77	48.17	45.71	46.91	44.28	47.02	44.9

## Data Availability

The data presented in this study are available on request from the corresponding author.

## Supplementary Materials

The following supporting information can be downloaded at: https://www.mdpi.com/article/10.3390/insects13030291/s1, Figure S1: Statistics on body length of silkworms between the AD group and ML group; Figure S2: Phenotype observation of silkworm fed with fresh mulberry leaves (ML) and artificial diet (AD); Figure S3: Volcano plot of DEGs between midgut and head; Figure S4: Expression level of the *Hex-t2* gene was measured by FPKM value; Figure S5: KEGG enrichment of FG-ADvsMG-AD, FG-MLvsMG-ML, FH-ADvsMH-AD and FH-MLvsMH-ML; Table S1: List of The Primers Used in This Study.
