# Supplementary material for: Midgut and Head Transcriptomic Analysis of Silkworms Reveals the Physiological Effects of Artificial Diets
Source: Insects. 2022 Mar 15;13(3):291. doi: 10.3390/insects13030291 (PMC8948783; doi:10.3390/insects13030291)
Supplement: Supplementary file 1 [file insects-13-00291-s001.zip › insects-1598737-supplementary.pdf]

**Supplementary Table S1: List of The Primers Used in This Study**

| Gene ID | Primer Name | Primer Sequences(5'-3') | Purpose |
|---|---|---|---|
| KWMTBOMO09651 | qpcr-PGRP-F | GGACCAGCCAATAAATTGAGCATCG | qPCR |
| KWMTBOMO09651 | qpcr-PGRP-R | CATTCTGTCGTCATAGCCTGATTGT | qPCR |
| KWMTBOMO10600 | qpcr-CYP450-F | CCCCACGTATCCTGACAGCAAACAC | qPCR |
| KWMTBOMO10600 | qpcr-CYP450-R | TAGGTCCAGTCCCGAATGGCAAGTA | qPCR |
| KWMTBOMO06450 | qpcr-betaGRP-F | TAACCGATAATGCAGAAGTCAACGA | qPCR |
| KWMTBOMO06450 | qpcr-betaGRP-R | GTGGCAAAATAAGAGGACCAGAAGC | qPCR |
| KWMTBOMO05616 | qpcr-LEB3-F | TATCAAGGTGACAATGTTCCTCGTG | qPCR |
| KWMTBOMO05616 | qpcr-LEB3-R | TATGATGTGAATGTTCAATGGACGC | qPCR |
| KWMTBOMO03709 | qpcr-Attacin-F | GAGTGTAGCGTTGTTGTTGTTGTGC | qPCR |
| KWMTBOMO03709 | qpcr-Attacin-R | GTACGTTCTTGTCGTTGCCGGTGAG | qPCR |

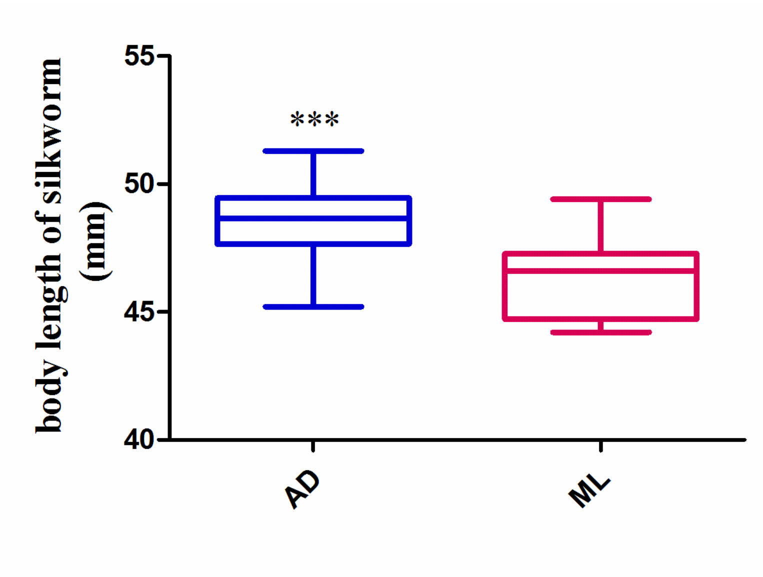


**Supplementary Figure S1.** Statistics on body length of silkworms between the AD group and ML group. Twenty silkworm larvae on day 3 of the fifth instar were randomly selected from the AD group and the ML group, respectively, and measured the body length. AD, artificial diets; ML, mulberry leaves. The "mm" means millimeter. The asterisks indicates a difference in significance (****p*-value < 0.001).

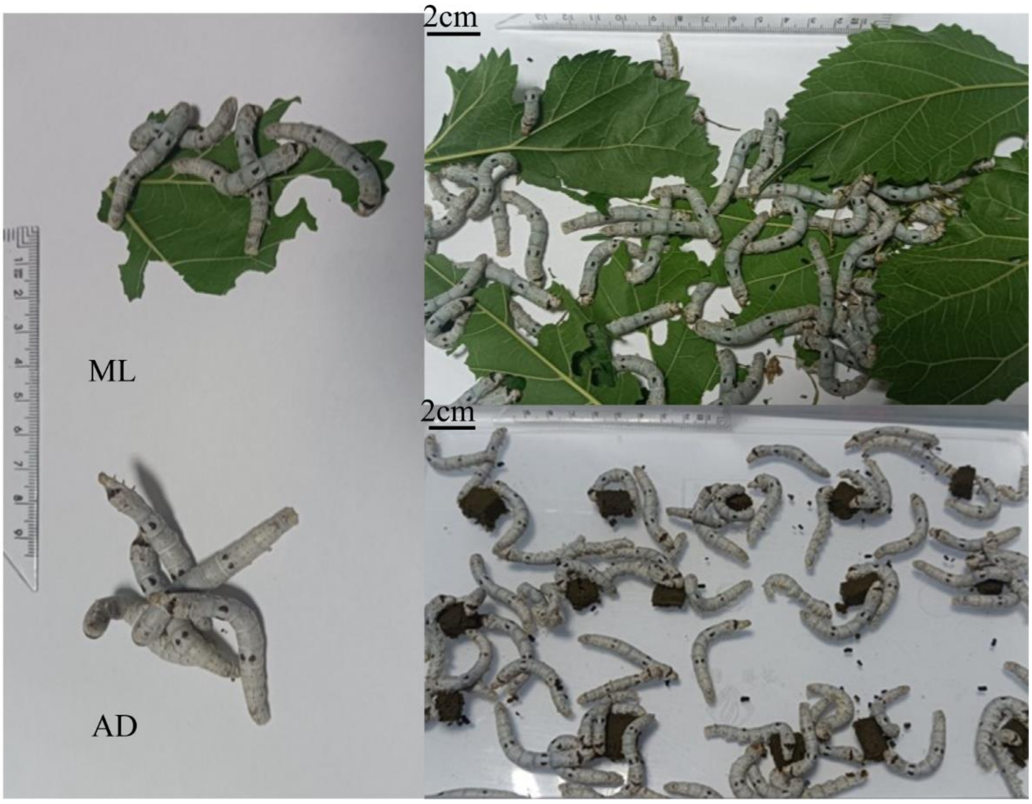


**Supplementary Figure S2.** Phenotype observation of silkworm fed with fresh mulberry leaves (ML) and artificial diet (AD).

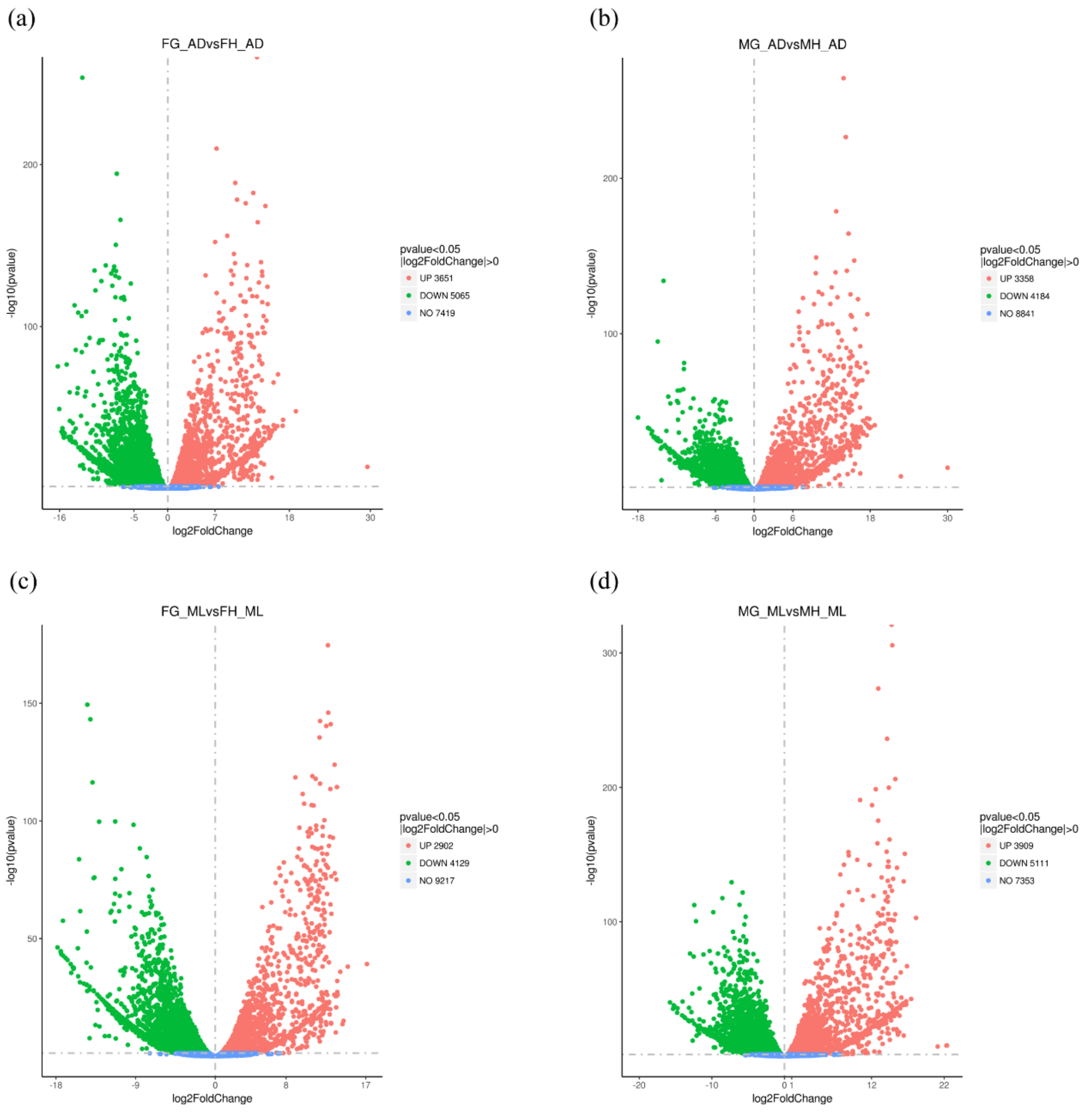


**Supplementary Figure S3.** Volcano plot of DEGs between midgut and head. Volcano plot for pair wise comparison for (A) FG_ADvsFH_AD, (B) MG_ADvsMH_AD, (C) FG_MLvsFH_ML and (D) MG_MLvsMH_ML. Horizontal oridinate represents the fold chang of gene expression, and the vertical ordinate represents the statistical significance of the change. Number of DEGs expressions (normalized to Log2 fold change) are displayed on the right side of each picture based on a P-adj cut-off of <0.05. The red plot represents a differentially upregulated gene, a green plot represents a differentially downregulated gene, and the blue plot means a gene that does not show differential expression.

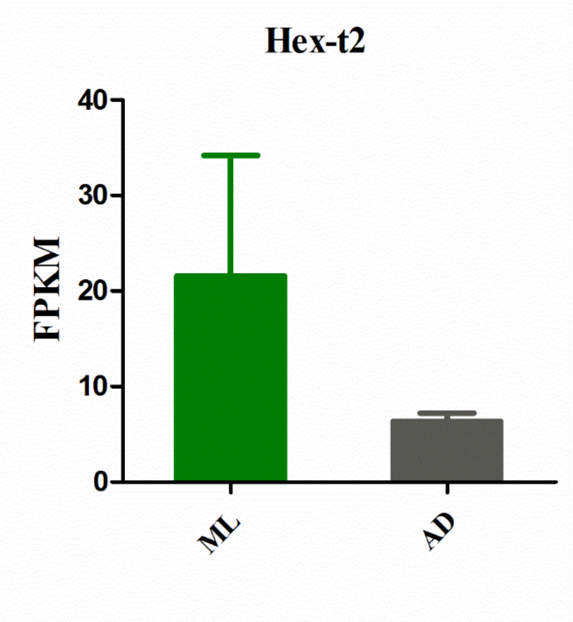


**Supplementary Figure S4.** Expression level of the *Hex-t2* gene was measured by FPKM value. ML represented the control group and AD represented the experimental group. Female represented the female silkworms and male represented the male silkworms.

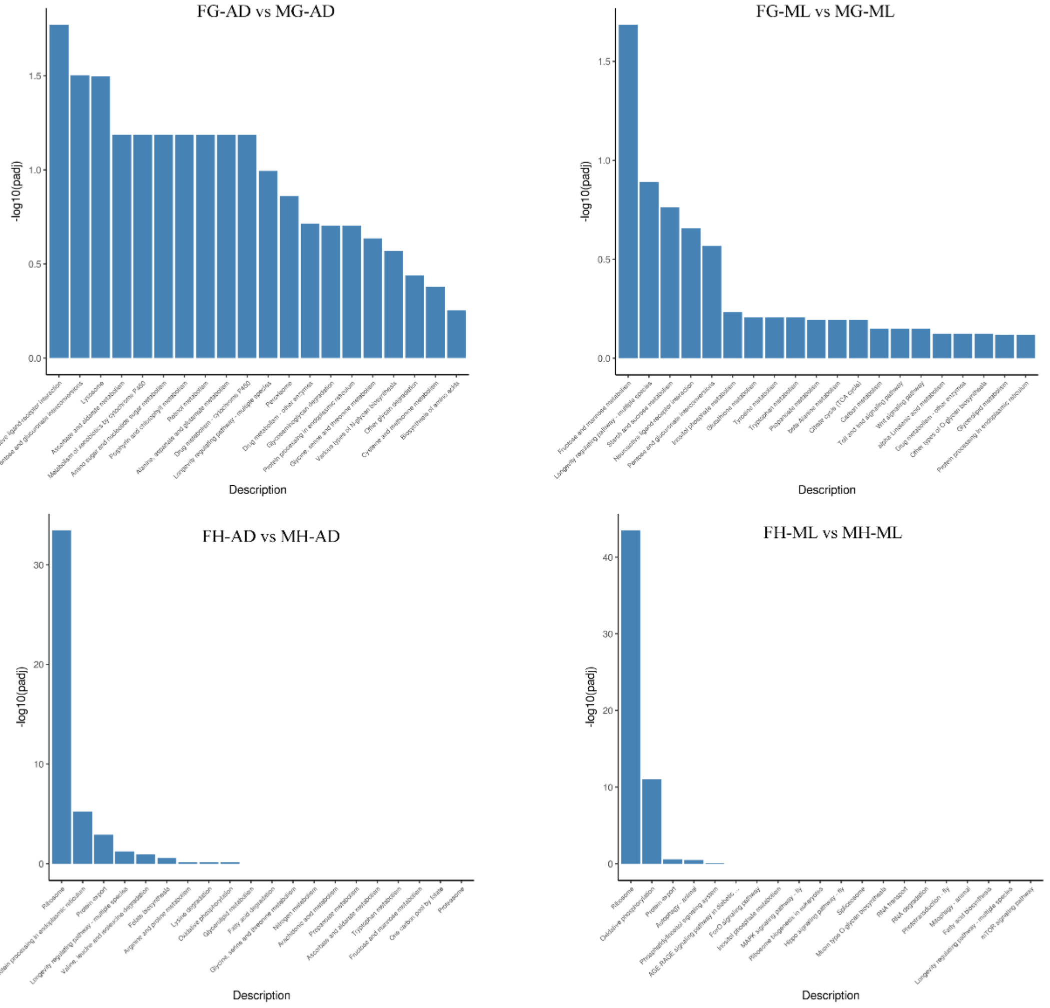


**Supplementary Figure S5.** KEGG enrichment of FG-ADvsMG-AD, FG-MLvsMG-ML, FH-ADvsMH-AD and FH-MLvsMH-ML. Statistics of top-20 enriched pathways of differentially expressed genes in each pairwise.
